# The comparison of electroencephalography power and event related potential in success and failure during multitask game

**DOI:** 10.3389/fnbot.2022.1044071

**Published:** 2022-11-16

**Authors:** Fumiya Sanuki, Nonthaporn Nakphu, Ayumi Tahara, Keiji Iramina

**Affiliations:** ^1^Graduate School of Systems Life Sciences, Kyushu University, Fukuoka, Japan; ^2^Department of Informatics, Faculty of Information Science and Electrical Engineering, Kyushu University, Fukuoka, Japan

**Keywords:** EEG, multitask, ERP, VR, game, Go/No-Go task

## Abstract

The first game-based treatment for children with attention-deficit hyperactivity disorder (ADHD) was approved by the United States Food and Drug Administration (FDA) in 2020. This game was developed for use at home as part of everyday training and can be used along with one’s usual training plan. In this game, two tasks are performed in parallel: (1) a perceptual discrimination targeting task (response and not response and avoiding responding to sudden pop-up targets) and (2) a sensory-motor navigation task (players continuously adjust their location to interact with or avoid positional targets). However, the brain activity of people playing this game was not examined, and the immersive environment (3D virtual world) was not considered. Therefore, we aimed to develop a system to investigate brain activity using electroencephalography (EEG) during multitask gameplay in virtual reality (VR). In this experiment, we focused on the difference between the success and failure of the Go/No-Go task in a multitask game. We created a color discrimination task and a target tracking task in VR. The content of this game task was designed using previous multitask training. EEG and event data were recorded. Using event data, we can analyze the data in detail. We divided the trial types (Go and No-Go) and results (success and failure). We then compared the success and failure of each task. In the Go trial, the relative theta power in success at Fz was significantly higher than that of failure. However, no difference in power was observed in the No-Go trial. On the other hand, theta power was no different between success and failure in the other task. These results of the Go trial suggest that the participants were attentive to processing both tasks. Thus, it is possible that theta power in the frontal area 1 s before stimulation could predict the success or failure of the Go trial. On the other hand, the results of the No-Go trial may be due to the low number of No-Go failure trials and the fact that stimulus oversight is one of the factors for success.

## Introduction

Attention-deficit hyperactivity disorder (ADHD) is a common childhood behavioral disorder that affects approximately 5% of children. ADHD presents during neurodevelopment, with symptoms of hyperactivity, impulsivity, and/or inattention in childhood (Sayal et al., 2018). Psychopharmacological treatment (treatment with medicine) is ineffective in approximately 36% of patients with ADHD and can have serious side effects. Various studies have shown that video games are potentially effective for ADHD treatment and assessment (Peñuelas-Calvo et al., 2022). Video games, as a treatment tool, use cognitive training as an intervention to improve executive function, such as attention. Engagement during training has been reported to be high. However, some points, such as attractive interfaces and appropriate healthcare settings, can still be improved.

The first game-based treatment for children with ADHD was approved by the U.S. Food and Drug Administration (2020) and is called EndeavorRx. This game was developed for use at home as part of everyday training. In one study (Kollins et al., 2020), 348 children (aged 8–12) with ADHD were evaluated using the Test of Variables of Attention–Attention Performance Index (TOVA API). They were randomly assigned to the AKL-T01 (EndeavorRx) or digital control intervention. An iPad mini 2 tablet was used in this experiment. The AKL-T01 algorithm was designed to improve attention and related cognitive control processes with an adaptive and personalized high degree of difficulty. Two tasks were to be performed in parallel (multitasking): (1) a perceptual discrimination targeting task (response and not response and avoiding responding to sudden pop-up targets) and (2) a sensory-motor navigation task (players continuously adjust their location to interact with or avoid positional targets). The training was adapted in real time for each player. In the perceptual discrimination task, users should respond to or ignore the stimulus, so it is similar to the Go/No-Go task. In the sensory-motor navigation task, users adjust their character location. The experimental period was 28 days. The patients were instructed to use AKL-T01 or the control at home for five sessions per day (25 min/day), 5 days per week for 4 weeks. In within-group analyses, the change in the TOVA API score significantly improved with AKL-T01 but not with the control. The idea for EndeavorRx multitasking training and adaptive adjustment was adapted from Anguera et al. (2013), who created a video game, NeuroRacer, to enhance cognitive control, including sustained attention and working memory in older adults. NeuroRacer includes two tasks that need to be done in parallel; a “sign task” to measure the player’s perceptual discrimination ability and a “driving task,” which is a concurrent visuomotor tracking task. This study compared the results of training using a single task (sign-only or drive-only) and multitasking (parallel sign-and-driving task). They evaluated the results of training using delayed-recognition tasks with and without distraction for working memory and TOVA for sustained attention. Multitasking training can enhance sustained attention and working memory. In addition, multitasking training still had an effect on cognitive control in participants at the 6-month follow-up. They also compared the results of multitasking and electroencephalogram (EEG) activity between trained and untrained older adults. They found that trained older adults obtained better results in multitasking and had an enhanced midline frontal theta power and long-range coherence effect than younger adults. Both EndeavorRx and NeuroRacer used an adaptive staircase algorithm to determine the difficulty level of the game for each player to perform both tasks with approximately 80% accuracy. Each individual can play the game at a customized challenge level. However, both of the studies mentioned above did not study the players’ brain activity and behavior while playing the respective games and did not consider the immersive environment (3D virtual world). EEG activity can be used to evaluate disorders or the index of user state, such as workload, attention, and alertness (Frey et al., 2014; Debnath et al., 2021). In frequency band power analysis, an increase in theta power in the frontal area or a decrease in the upper alpha power indicates the mental process (Scharinger et al., 2020). In the Go/No-Go task, a researcher used the event-related potential (ERP) method for evaluating reactions toward stimuli (Perri et al., 2014). Dan and Reiner (2017) studied EEG-based cognitive load between 3D virtual worlds and 2D displays. The task that participants had to do was learn paper-folding (origami) by observing 2D or 3D displays. They recorded the power of the alpha and theta oscillations and calculated the cognitive load index (CLI). The results showed that there was a significantly higher CLI when processing the 2D projection than when processing the 3D projection. In addition, participants with lower spatial abilities benefited more from the 3D display than from the 2D display. Initially, video games had limited potential for immersion (Rubio-Tamayo et al., 2017). However, the influence of virtual reality (VR) on video games has led to them having a more immersive environment. VR and video games are used as research tools and improve people’s quality of life. VR can be useful in medical research and data visualization. We focused on brain activity while playing a multitasking game similar in structure to the multitasking games that have been shown to be effective in training ADHD patients and the elderly. In particular, we investigated the causes of task failure and brain activity during task failure. By targeting young people with relatively high cognitive ability, we investigated the relationship between the causes of rare failures and brain activity. This may allow us to infer failures and their causes from brain activity. In this experiment, we focused on the difference of brain activity between the success and failure of the Go/No-Go task in a multitask game.

## Materials and methods

### Participants

We recorded data from nineteen Kyushu University students (sixteen males and three females). Mean of their age was 23.6 years with a range of 19 to 29 years. They had normal or corrected-to-normal vision and were healthy participants. They were not a professional in the game. Before the experiment, all the participants provided written informed consent. This study was conducted in accordance with the ethical standards of the Declaration of Helsinki and approved by the ethics review board of Kyushu University.

### Equipment

#### Head-mounted display

**FIGURE 1:**
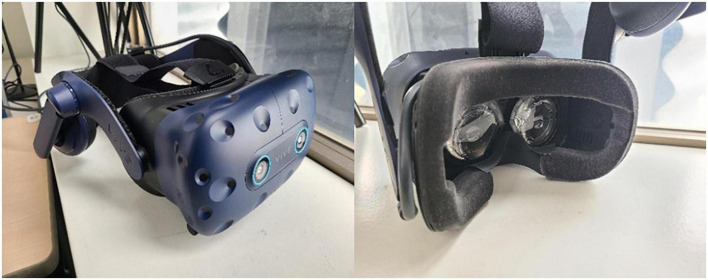
HTC VIVE pro eye.

HTC VIVE Pro Eye was used to display the game during the experiment. The HMD also included two controllers and two base stations of 2.0. The devices were connected *via* a link box, USB 3.0, and DisplayPort cables and connected to a computer. The screen in the VR headset was a dual OLED 3.5 inch diagonal with a resolution of 1,440 × 1,600 pixels per eye (2,880 × 1,600 pixels combined). The VR headset had a refresh rate of 90 Hz. The audio device, other support sensors, and ergonomics were integrated. An example of a VR headset is shown in Figure 1.

For controllers, StreamVR Tracking 2.0 was used as the sensor. The input was a trackpad, grip buttons, dual-stage trigger, system button, and menu button. They were chargeable using a MicroUSB port. In this study, one controller was used during the experiment. Base stations were used for detecting the headset. The room setup was checked before starting the experiment.

The HMD needed to be connected to a computer *via* StreamVR software. The HMD can be successfully installed following the official instructions.

#### Electroencephalogram

**FIGURE 2:**
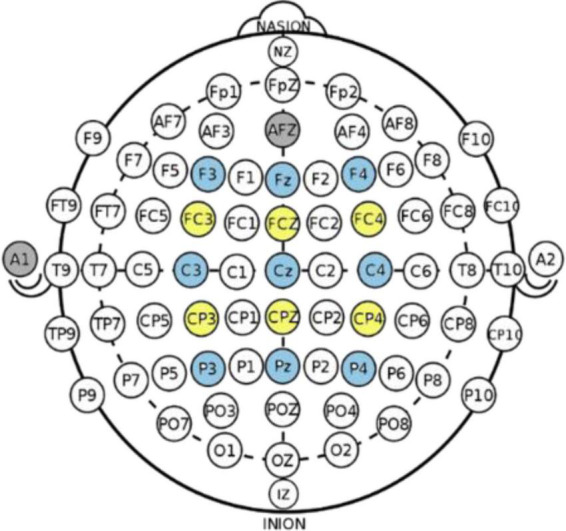
Electroencephalography (EEG) electrode.

Brain activity data were obtained using the g.USBamp by g.tec medical engineering company. MATLAB (R2017a) and Simulink were used to measure and record the data. Fifteen channels were used: F3, Fz, F4, FC3, FCz, FC4, C3, Cz, C4, CP3, CPz, CP4, P3, Pz, and P4 with the reference on the left ear, and the ground as AFz, as shown in Figure 2. The sampling rate of the EEG recordings was 512 Hz. Impedance was under 5 kohm.

#### Computer

The computer for the multitask game ran the game software (Unity) and connected to the HMD. This computer could be connected to a computer for EEG recording and to g.tec (g.GAMMAbox and g.USBamp) to receive brain activity data and send time data.

The game system was developed using Unity 2019.4.13f1. Unity could connect to the HMD *via* the StreamVR plug-in. EEG data were sent *via* UDP from another computer using the Simulink model (MATLAB R2017a).

### Game design

#### Color discrimination task

**FIGURE 3:**
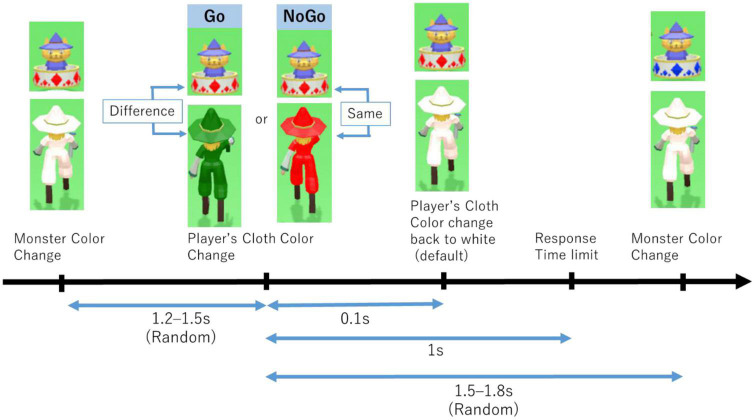
Color discrimination task flow.

The CDT is a task adapted from the Go/No-Go task (GNGT). GNGT is a classic task for measuring response inhibition and has been used by Kollins et al. (2020) and Peñuelas-Calvo et al. (2022). The task consisted of Go and No-Go trials. The task consists of continuous Go trials with rare No-Go trials suddenly appearing. No-Go trials in CDT were set to randomly appear at around 20%. In this task, you have to compare the color of two objects: the monster’s jewel and the character’s cloth. There were four color varieties: red, green, blue, and yellow. The color of the monster’s jewel changed every 3 s. The character’s cloth changed randomly 1.2–1.5 s after a change in the color of the monster’s jewel. However, the response time was less than the interval time (1 s). Participants had to respond when the colors of the monster’s jewel and the character’s cloth were not the same to obtain the score and decrease the monster’s health point (HP). If participants responded while they had the same color, their score would decrease, and the monster’s HP would increase. The flow of this task is illustrated in Figure 3. The monster’s color is different from Figures 3, 5. This change occurs after the monster is defeated. In other words, when the new monster appeared, the monster’s color changed from the previous one.

We recorded five main events during this task: response to correct color or Go success event, no response to correct color or Go failure event, avoid responding to the wrong color (AR) or No-Go success event, respond to wrong color or No-Go failure event, and defeated a monster. These events were used for the score and monster’s HP calculation. The task results were used for the accuracy calculation, as shown in Table 1. To respond to each trial of this task, participants had to pull the trigger on the controller as fast as possible after the color of the character’s cloth had changed. In addition, we could record changes in the color of the character’s cloth and the color of the monster’s jewel.

**TABLE 1:** Score and monster’s HP calculation for each event in the CDT (upper) and TTT (lower).

Event	Score	Monster’s HP
Go (Success)	+10	−20
Go (Failure)	−5	
NoGo (Success)	+5	
NoGo (Failure)	−10	+10
Get target	+5	
Miss target	−1	
Avoid obstacle	+5	
Hit obstacle	−5	

#### Target tracking task

**FIGURE 4:**
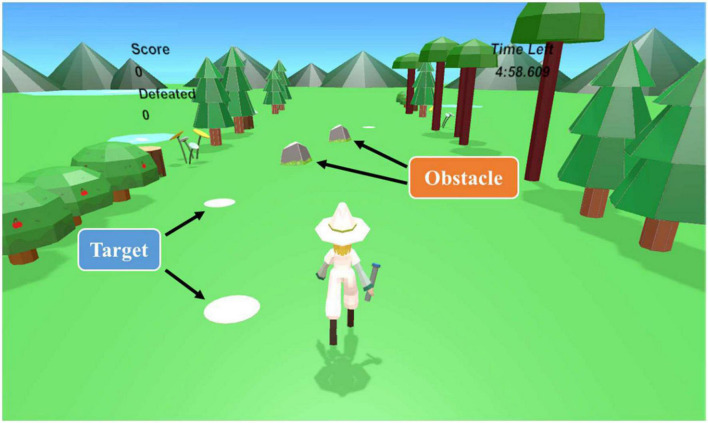
Example of the target tracking task.

This task aims to train visuospatial function, which involves the perception, recognition, and manipulation of visual stimuli (Choi et al., 2020). In the TTT, participants had to control the character’s movement to the left or right while the character was moving forward to get the target and avoid the obstacle to get the score. If the character misses the target or hits an obstacle, the score decreases. The target and obstacle randomly appeared in one of the five lanes of the road. The percentage appearance of the obstacle was 20%, and the obstacle does not appear more than twice in a row. The objects randomly appeared in the closest lane at a fixed distance, depending on the task level. Four events were detected in this task: get the target, miss the target, avoid the obstacle, and hit the obstacle. These events were used for the score and accuracy calculations, as shown in Table 1. The participants clicked the trackpad on the controller to control the movement of the character. An example of this task is shown in Figure 4.

**FIGURE 5:**
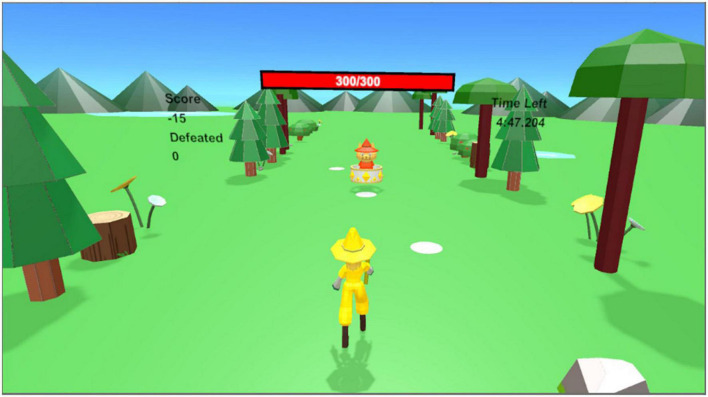
Example of multitasking (Combine CDT and TTT).

When the level changes, the speed of the character moving forward will change; that is, the time interval to reach each object will change. There were 55 levels for this task. At level 0, the time interval for reaching each object was 3,333 ms. The highest TTT level was 54, with an interval of 286 ms.

#### Multitask

This task consists of a combination of TTT and CDT. An example of this task is shown in Figure 5. The rule of each task is same. So, participants need to pay attention to targets, obstacles, the color of the monster’s jewel and the character’s cloth and control the character.

#### Adaptive level adjustment

**FIGURE 6:**
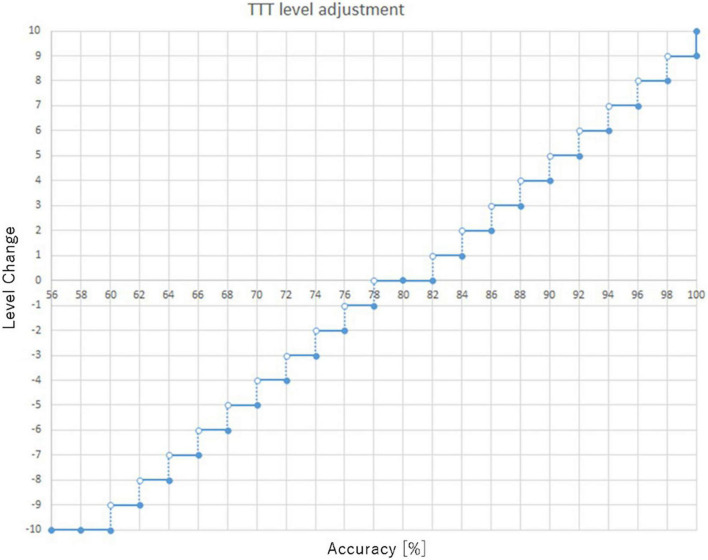
Target tracking task (TTT) level adjustment.

The adaptive level adjustment was adapted from Kollins et al. (2020) and used the staircase algorithm. The level of each task was adjusted by accuracy every 1 min. If the accuracy was 80%, the level did not change. However, for TTT, the level increased by one if the accuracy was more than 82%. For example, if the TTT accuracy was 90%, the TTT level was increased by four levels, as shown in Figure 6.

### Electroencephalogram analysis

The multitask game was played for 30 min. We selected the CDT in the multitask data. We used MATLAB (R2016b, The MathWorks, Natick, MA, USA) and EEGLAB (14_1_2b, Swartz Center for Computational Neuroscience) for EEG analysis. For pre-processing, a finite impulse response (FIR) filter (1–30 Hz) was applied, and noisy epochs were visually deleted. We applied an independent component analysis to remove the components assumed to be derived from eye movement and blinking. In addition, we calculated the ERP using averaging. We used from −500 ms to 1,000 ms after changing the character’s color and subtracted the average from −500 ms to 0 ms as the baseline from the averaged data. We calculated the relative power (RP) for 1 s using fast Fourier transform (FFT) before changing the character’s color. We calculated the theta power (4–7 Hz), alpha power (8–13 Hz) and beta power (15–30 Hz).

Relative power means the percentage of power in power in all frequency bands. The equation RP is given by


$$R\,P=\sum_{i=k}^{n}p\,o\,w\,e\,r\,\left(i\right)/\sum_{j=1}^{m}p\,o\,w\,e\,r\,\left(j\right)$$

where i and j are specific frequencies, m is the maximum frequency of all frequency bands, k and n are specific frequency bands (e.g., Using theta bands, we used *k* = 4 and *n* = 7) and power(i) and power(j) is power at i Hz and j Hz.

In each dataset, we divided Go and No-Go into success and failure. Success and failure in the Go trial were defined as pushing or not the button within the interval time. Failure in the No-Go trial was pushing the button within the interval time. In addition, we checked TTT accuracy 1 s before and after changing the character’s color. Therefore, we divided TTT into success and failure.

### Statistical analysis

We used Wilcoxon’s signed-rank test to compare the RP of success and failure in the Go/No-Go trial and in the TTT 1 s before and after changing the character’s color. We used the Kruskal–Wallis test to compare the accuracy of TTT in 1 s before and after changing the character’s color for success and failure in the Go/No-Go trial. In these tests, a *p*-value of < 0.05 was considered statistically significant. We used Mann-Whitney *U*-test and the Bonferroni’s correction as multiple comparisons. In this test, a *p*-value of < 0.0083 = 0.05/6 was considered statistically significant.

## Results

### Behavior

**FIGURE 7:**
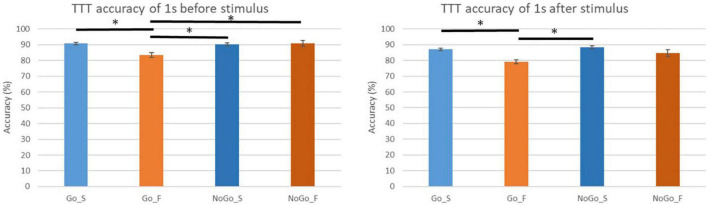
Target tracking task (TTT) accuracy of 1 s before and after changing the character’s color in each conditions. *p < 0.0083.

This game was played for 30 min. It is about 600 trial of Go/No-Go task. Table 2 show number of trials used to EEG analysis. This number show number of trial after rejecting noisy epochs. We calculated TTT accuracy of 1 s before and after changing the character’s color in each conditions. Figure 7 shows averaged TTT accuracy in each condition. The left figure shows averaged TTT accuracy of 1 s before changing the character’s color in each condition. The right figure shows averaged TTT accuracy of 1 s after changing the character’s color in each condition. In TTT accuracy of 1 s before stimulus, we found significant differences between conditions (*p* = 0.0014 < 0.05). We found TTT accuracy of success in the Go trial was significantly higher than that of failure in the Go trial (*p* = 0.00033 < 0.0083). TTT accuracy of success in the NoGo trial was significantly higher than that of failure in the Go trial (*p* = 0.0015 < 0.0083). TTT accuracy of failure in the NoGo trial was significantly higher than that of failure in the Go trial (*p* = 0.0079 < 0.0083). On the other hand, In TTT accuracy of 1 s after stimulus, we found significant differences between conditions (*p* = 0.000010 < 0.05). We found TTT accuracy of success in the Go trial was significantly higher than that of failure in the Go trial (*p* = 0.0000079 < 0.0083). TTT accuracy of success in the NoGo trial was significantly higher than that of failure in the Go trial (*p* = 0.0000079 < 0.0083).

**TABLE 2:** Number of trials used to EEG analysis.

	Go_S	Go_F	NoGo_S	NoGo_F	All trial
Average	388.1	68.9	97.8	16.9	571.8
Standard error	10.4	8.7	3.8	2.2	8.2
Participant 01	344	124	102	25	595
Participant 02	342	113	73	36	564
Participant 03	402	64	114	9	589
Participant 04	456	18	77	22	573
Participant 05	299	81	94	7	481
Participant 06	411	84	98	7	600
Participant 07	468	5	107	17	597
Participant 08	425	45	118	5	593
Participant 09	421	66	90	20	597
Participant 10	366	95	104	14	579
Participant 11	397	26	98	23	544
Participant 12	363	128	61	35	587
Participant 13	375	64	98	11	548
Participant 14	450	5	110	20	585
Participant 15	339	52	74	16	481
Participant 16	418	57	120	4	599
Participant 17	346	120	99	26	591
Participant 18	377	84	104	14	579
Participant 19	375	79	118	10	582

### Electroencephalogram

#### Event-related potential

**FIGURE 8:**
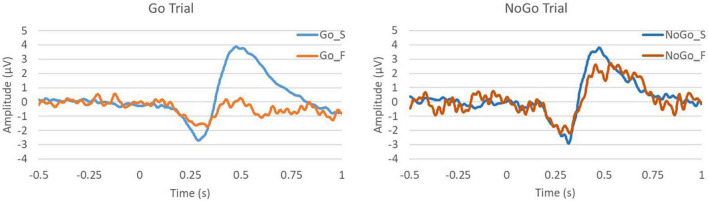
The ERP at Cz in the Go and No-Go trials.

Electroencephalogram data were obtained from all participants and were analyzed in MATLAB. Using event data, we could look at ERP based on the timing of the color change of the character’s cloth in the CDT task. Furthermore, by dividing tasks into Go and No-Go trials and success and failure cases, we could evaluate the differences in trends. Figure 8 shows the ERP of the Go and No-Go trials to compare success and failure. This figure shows the time on the horizontal axis and the amplitude on the vertical axis. Furthermore, 0 s is the start time of the trial, that is, the time when the character’s cloth color changes. The blue line represents success, and the orange line represents failure. In the ERP of success in Go and No-Go trial and failure in No-Go trial, we observed a negative peak of N200 after stimulation, followed by a positive peak of P300.

#### Relative power

**FIGURE 9:**
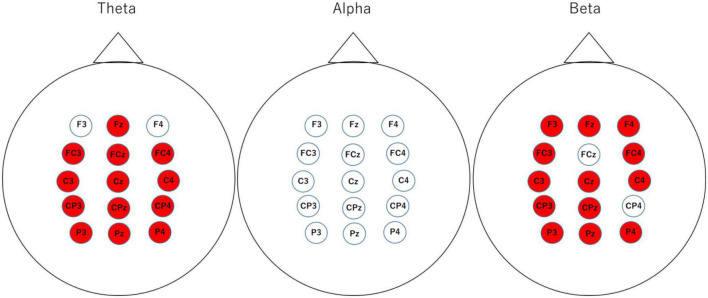
The averaged relative power of theta, alpha and beta at all channels in 1 s before the stimulus in the Go trials.

Figure 9 shows the map of the averaged RP of theta, alpha and beta at all channels in 1 s before the stimulus in the Go trials. The red color means the power of success is significantly higher than that of failure (*p* < 0.05).

**FIGURE 10:**
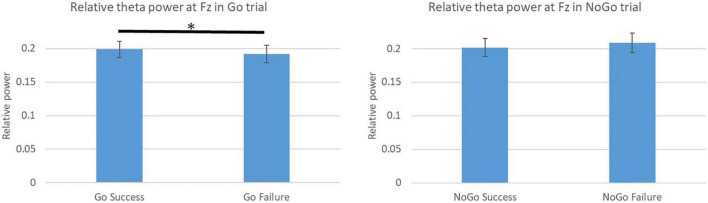
Relative theta power at Fz 1 s before the stimulus. *p < 0.05.

**FIGURE 11:**
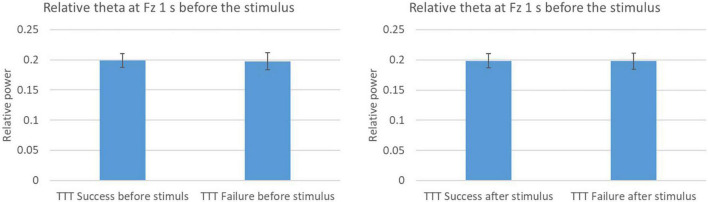
Relative theta power at Fz by dividing result of TTT.

Figures 10, 11 show the RPs under certain conditions. We compared the power of success or failure using event data. Figures 10, 11 show the average relative theta power in 1 s before the stimulus in the Go and No-Go trials. Figure 10 shows the difference between success and failure in the Go and No-Go task. In the Go trial, the relative theta power in success was significantly higher than in failure (*p* = 0.028 < 0.05). Success in the Go trial meant the participant could pull the trigger on the controller before the time limit. Failure in the Go trial meant that the participant could not pull the trigger on the controller in the trial. Success in the No-Go trial meant that the participant was able to avoid pulling the trigger on the controller within the time limit. Failure in the No-Go trial means that the participant could not avoid pulling the trigger on the controller in the trial.

Figure 11 shows the difference between success and failure in TTT of 1 s before and after changing the character’s color. Success in the TTT meant the participant get the target and avoid the obstacle. Failure in the TTT meant the participant miss the target or hit the obstacle. There were no significant differences between success and failure in 1 s before and after the stimulus.

## Discussion

In this study, the participants’ brain activity during gameplay was assessed. We aimed to analyze participants’ brain activity by dividing success and failure during gameplay. For the CDT task, we compared ERP and RP by dividing them into Go/No-Go trials of success and failure. In ERP success, we observed N200 and P300 in response to the stimulus. First, P300 in the ERP is known to be a response to stimuli (Berchicci et al., 2012). P300 was seen with success in the Go trial and in the No-Go trial and failure in the No-Go trial, whereas it was not seen with failure in the Go trial. These results suggest that the participants were unaware of the CDT stimuli in the failure of the Go trial.

Based on this result, we focused on the difference in power depending on the success or failure of the Go trial. Therefore, we compared the power at all electrodes with the success or failure of the Go trial. The results showed that there was a significant difference in power for a wide range of theta and beta, but not for alpha power. Beta power has been associated with tasks that require arousal and perception (Llamas-Alonso et al., 2019). It is also said to be associated with better multitasking performance and increased Beta (Hsu et al., 2017). Therefore, it was suggested that during the preparation phase of the Go trial, Beta power may be higher when two tasks can be handled simultaneously and lower when they cannot be handled well. Attention and concentration were related to theta power in the frontal area and alpha power in the parietal area. In particular, the theta power of the frontal area is said to be higher during states of high attention and concentration (Llamas-Alonso et al., 2019). On the other hand, the alpha power of the parietal area is said to be higher when attention is reduced (Clayton et al., 2015; Shou et al., 2015). Other studies of multitasking have shown that brain activity during multitasking increase the theta power in the frontal area and power in the parietal area as the number of tasks being processed simultaneously increases. The other research reported that alpha power increase in the posterior area due to the suppression of unrelated stimuli from the environment (Ward, 2003). Furthermore, the alpha power in the parietal area is said to be unsystematic (Puma et al., 2018). In this study, the results of power also indicate that theta power in the frontal area was higher during success before the task than during failure. These results suggest that participants were attentive to processing both tasks in the successful trial, and they paid attention to processing only one of the tasks in the failed trial. On the other hand, alpha power in the parietal area was no significant difference between success and failure. It seems that this result was caused by a combination of various factors. These results suggest that differences in theta power could be seen during the preparation phase of one of the tasks and during the processing of the task. Thus, it is possible that theta power in the frontal area 1 s before stimulation could predict the success or failure of the Go trial. However, no difference in power was observed in the No-Go trial. This may be due to the low number of failures in the No-Go trial and the fact that stimulus oversight was one of the factors for success in the No-Go trial. In the future, we believe that eye tracking data will allow us to compare successes and misses separately by examining whether the participants were looking at the stimulus when they succeeded in the No-Go trial.

Based on these results, we focused on the difference in frontal theta power depending on the success or failure of the Go trial. Therefore, we examined the differences in power due to TTT success or failure and TTT correctness in the 1 s before and after the CDT to consider whether TTT success or failure had an effect in the 1 s before and after the CDT. First, the results of the TTT correct response rate in the 1 s before and after the CDT showed that the TTT correct response rate was significantly lower before and after the failure of the Go trial than in the other conditions. In other words, it is thought that attention to the TTT was lower during the failure of the Go trial than during the other conditions. On the other hand, the results of the difference in power due to TTT success or failure showed that the difference in frontal theta power due to TTT success or failure was not significant. These results suggest that there was no relationship between TTT success or failure and frontal theta power and that frontal theta power differed depending on success or failure in the Go trial. Therefore, it seems that theta power during the preparation phase represents the attention to the Go trial.

## Data availability statement

The raw data supporting the conclusions of this article will be made available by the authors, without undue reservation.

## Ethics statement

The studies involving human participants were reviewed and approved by the ISEE in Kyushu University. The patients/participants provided their written informed consent to participate in this study.

## Author contributions

FS and KI contributed to the conception and design of the study. FS analyzed the data and wrote the first draft of the manuscript. AT and NN made multitask game for this study. All authors contributed to the article and approved the submitted version.
